# Effect of Dietary Incorporation of Yellow Mealworm as a Partial Fishmeal Replacer on Growth, Metabolism, and Intestinal Histomorphology in Juvenile Meagre (*Argyrosomus regius*)

**DOI:** 10.1155/2023/6572421

**Published:** 2023-06-24

**Authors:** Margarida Saavedra, Marisa Barata, Ana Catarina Matias, Ana Couto, Ahmed Salem, Laura Ribeiro, Teresa Gama Pereira, Margarida Gamboa, Cátia Lourenço-Marques, Florbela Soares, Jorge Dias, Pedro Pousão-Ferreira

**Affiliations:** ^1^Division of Aquaculture and Upgrading, Portuguese Institute for the Sea and Atmosphere, I.P (IPMA), Rua Alfredo Magalhães Ramalho, N°6, 1495-006 Lisboa, Portugal; ^2^Interdisciplinary Centre of Marine and Environmental Research (CIIMAR), University of Porto, Terminal de Cruzeiros do Porto de Leixões, Av. General Norton de Matos, s/n, 4450-208 Matosinhos, Portugal; ^3^MARE-Marine and Environmental Sciences Centre & ARNET-Aquatic Research Network Associated Laboratory, NOVA School of Science and Technology, NOVA University of Lisbon, Portugal; ^4^Aquaculture Research Station of IPMA, Parque natural da Ria Formosa, s/n, 8700-194 Olhão, Portugal; ^5^S2AQUA-Collaborative Laboratory, Association for a Sustainable and Smart Aquaculture, Av. Parque Natural da Ria Formosa s/n, 8700-194 Olhão, Portugal; ^6^Department of Biology, Faculty of Ciências, University of Porto, Rua do Campo Alegre, 4169-007 Porto, Portugal; ^7^National Institute of Oceanography and Fisheries (NIOF), Egypt; ^8^Sparos Lda, Área Empresarial de Marim, Lote C, 8700-221 Olhão, Portugal

## Abstract

Efforts have been made to find alternatives to fish meal (FM), as the sustainability of aquaculture depends on it. Insect meal (IM) is a potential candidate to partially replace FM, being more sustainable and economically viable. In this experimental trial, three diets were tested with different yellow mealworm incorporation: a control diet with no IM, a diet with an inclusion of 10% IM (Ins10), and a diet with an incorporation of 20% IM (Ins20). The diets were tested on 10.5 g meagre for 47 days. The results showed that an IM inclusion higher than 10% affected both growth (2.6 vs. 2.2) and FCR (1.5 vs. 1.9) of meagre juveniles. However, this reduction in growth did not result from lower protein retention or changes in muscle fibre area or density. Little differences were observed in the activity of pancreatic and intestinal enzymes except for aminopeptidase total activity which was higher in the control and Ins10 compared to Ins20 (3847 vs. 3540 mU/mg protein), suggesting no limitations in protein synthesis. Also, the alkaline phosphatase intestinal maturation index was higher in the control group compared to the IM groups (437 vs. 296). On the contrary, several differences were also found in the proteolytic activity in the hepatic and muscle tissues of meagre juveniles fed the Ins10 diet. The inclusion of IM had no impact on intestine histomorphology but changes were detected in the enterocytes of fish from control and Ins10 which showed hypervacuolization and nucleus misplacement compared to the Ins20 treatment. Nevertheless, a higher percentage of *Vibrionaceae* was recorded for meagre fed on the Ins20 diet. Since no signs of inflammation were observed in the distal intestine, this suggests IM incorporation could have had an important impact on intestinal health due to its antimicrobial properties. This is supported by an increase in the haematocrit in the treatments where IM was added (20 to 25%). In conclusion, incorporations of IM at percentages up to 10% do not seem to have a negative impact on meagre performance at this age but can enhance the fish immune system and protection against intestinal inflammation.

## 1. Introduction

Finding suitable fish meal (FM) replacers has been an important challenge in aquaculture, as it is difficult to find alternatives that do not negatively affect either growth or fish performance [1–3]. Up to now, most feeds rely mainly on fish meal (FM), supplied from wild fish, which is associated with several issues such as wild population depletion and higher production costs.

Insect meal (IM) is a strong candidate for FM partial replacement, showing an interesting nutritional composition, especially in terms of protein content and amino acid profile [4], and, above all, a high potential in terms of sustainability. Insect production is not complex, does not require a large space, insects feed is cheap, and its carbon footprint is much lower compared to other meat industries [5–7]. Seven insect species have been authorized by the European Union to be included in fish diets (EC Regulation 893/2017; European Commission, 2017). From these, two species have shown the most promising results: black soldier fly (*Hermetia illucens*) and yellow mealworms (*Tenebrio molitor*) [8, 9]. For both species, there have been reported variations in nutrient composition, a result of its high dependence on substrates and processing methods [10]. In general, yellow mealworms have higher crude protein content (47-66% vs. 36-59% for mealworms and black soldier fly, respectively) [11–13].

Several studies have been carried out in different fish species to test FM replacement by insect meal [14–17]. In general, IM incorporation at moderate levels does not negatively affect fish growth. However, when high incorporation levels are used, fish growth and performance can be affected, depending on the insect species used [10]. This is more evident for black the solider fly than for the yellow mealworm [10]. In fact, some studies report successful incorporations of up to 75% of *T. molitor* (TM) in the yellow catfish diet without compromising growth [18]. In other studies, although growth was not affected, fillet quality changed, especially the fatty acid profile [8]. The percentage of incorporation can be maximised using a defatted meal or an insect meal enriched in n-3 long chain fatty acids, with inclusion rates up to 50% without restraints on growth [19], or the fatty acid composition of the fillet [20].

Meagre is a carnivorous species with a high protein requirement [21]. Several studies have been carried out with meagre using different levels of IM incorporated into the diet as an alternative to FM [14–16, 22]. However, the results from the different studies are not clear on the acceptable level of IM incorporation for this species. This study aimed at clarifying the meagre response to dietary incorporation of yellow mealworms (0, 10, and 20%), especially on juvenile growth, survival, and metabolism, by integrating physiological information on pathways linked to digestion and protein metabolism.

## 2. Material and Methods

### 2.1. Experimental Diets

Three different diets were tested: a control, without yellow mealworm, a diet with 10% yellow mealworms (Ins10), and a third diet with 20% yellow mealworms (Ins20). The diets were formulated and produced by Sparos Lda (Olhão, Portugal). The production protocol is described in Saavedra et al. [23]. The diets were crumbled (Neuero Farm, Germany) and sieved to 1.5 and 2 mm. The formulation of the three diets is presented in Table 1.

### 2.2. Experimental Set-up

This study took place at the Aquaculture Research Station of the Portuguese Institute for the Sea and Atmosphere (IPMA, I.P.) and was carried out in compliance with Directive 2010/63/EU, and institutional guidelines for the care and use of animals were followed by the authors.

Meagre juveniles aged 74 days with an initial weight of 10.5 ± 1.2 g and 10.2 ± 0.4 cm total length were placed in nine 250 L fibreglass raceways, each containing 80 fish. An open-water system was used, and, during the experimental trial, oxygen and temperature were 5.1 ± 0.8 mg/L and 23.2 ± 2.9°C, respectively, while salinity was 38 ± 1 ppt. The photoperiod was 14 L/10 D. The experimental trial lasted 47 days, and each experimental feed was tested in triplicate tanks (*n* = 3).

### 2.3. Sampling and Biochemical Analysis

At the beginning of the trial, 115 fish were individually weighed and measured, and 25 fish were collected to determine fish proximal composition. Five weeks after the trial started, a midterm sampling was carried out to evaluate fish growth, and 50 fish were sampled (weighed and measured). When the experimental trial was over, fish biometrics from all tanks were taken. Ten fish per tank were used to analyse whole body composition (in pools), six fish per tank were anesthetised (700 ppm 2-phenoxyethanol) [24], and blood was drawn from the caudal vein. These fish were immediately euthanized and used to dissect different tissues for further analysis such as the anterior intestine, used for quantifying the digestive enzymes, and the terminal portion of the gut, for microbiota and histological analysis. A cross-section of the first dorsal ray was cut for muscle cellularity analysis, and, for the proteolytic analysis, the white muscle and liver from nine fish per treatment were taken. Samples for microbiota analysis were processed immediately, samples for histological techniques were fixed in specific solutions; samples for body composition analysis were frozen at -20°C; and whereas samples for enzyme and protein metabolism analysis were flash frozen in liquid nitrogen and stored at -80°C.

### 2.4. Analytical Component

#### 2.4.1. Proximate Composition

The composition of diets and whole-body was determined in duplicate following the AOAC procedures [25]. The pool of ten fish per tank (thirty per treatment) was freeze-dried first, with the exception of samples for moisture and ash analysis. All analyses, including crude protein and lipid, were carried out according to Saavedra et al. [23].

#### 2.4.2. Haematocrit and Lipidic Peroxidation (LPO) on Blood Components

The haematocrit (HCT), expressed as a percentage of total blood volume, was quantified by microhaematocrit capillaries filled with blood and centrifuged (EBA 21 Hettich) at 5000 g for 5 min.

Red blood cells (RBCs) are susceptible to lipid peroxidation because of their function as oxygen carriers and their lipid composition. Malondialdehyde (MDA) concentration, a marker, was used to quantify the peroxidation levels in the red blood cells by reacting with thiobarbituric acid, producing coloured thiobarbituric acid-reacting substances (TBARS) that were measured. The results were expressed as nanomoles of MDA per gram of total protein.

#### 2.4.3. Digestive Enzymes Activities

To determine the digestive enzymes activity, intestine homogenates were used. Purification of the brush border membranes followed the protocol described in Ribeiro et al. [26]. Briefly, intestinal segments were homogenized in 30 volumes (w/v) of ice-cold manitol (50 mM), Tris-HCl buffer (2 mM), pH 7.0, at a maximum speed of 30 s (IKA homogeneizer) to obtain the brush border membranes, according to Crane et al. [27]. Later, 100 *μ*l of 0.1 M CaCl 2 was added. The homogenate was then centrifuged at 9000 g at 4°C for 10 min, followed by the collection of the supernatant, which was centrifuged again at 34000 g at 4°C for 20 min. The pellet was re-suspended in Tris-Hepes DTT buffer and used to quantify enzyme activity.

Trypsin (E.C.3.4.21.4) activity was determined at 25°C using BAPNA (N*α*-Benzoyl-DL-arginine-p-nitroanilide) as substrate in trizma-CaCl2 buffer (20 mM), pH 8.2, by the method from Holm et al. [28]. Amylase (E.C.3.2.1.1) activity was assayed using starch dissolved in NaH2PO4 buffer (0.07 M), pH 7.4 [29], while alkaline phosphatase (E.C.3.1.3.1) activity was assayed using pNPP 5 mM (p-nitrophenylphosphate) in a solution of carbonate buffer (30 mM), pH 9.8 [30]. Aminopeptidase N (E.C.3.4.11.2) activity was quantified using L-leucine p-nitroanilide (0.1 M), as substrate, in buffer phosphate (80 mM), pH 7.0, following Maroux et al. [31] protocol. Acid phosphatase (E.C.3.1.3.2) activity was analysed according to Terra et al. [32] using pNPP 5.5 mM (p-nitrophenylphosphate) in a solution of citrate buffer 0.1 M (citric acid and sodium citrate), pH 4.8. Enzyme activity was calculated as micromoles of substrate hydrolysed per minute (i.e., U) at 37°C for alkaline phosphatase and aminopeptidase and at 25°C for trypsin. Amylase activity was expressed as the activity required to hydrolyse 1 mg of starch in 30 min at 37°C.

Protein was quantified by the Bradford method [33]. Enzyme activity was expressed as specific activities, i.e., U/mg protein and total activity per U/segment. Enterocyte maturation index was calculated as the ratio between brush border enzyme total activity (alkaline phosphatase and aminopeptidase) and acid phosphatase, according to Zambonino-Infante et al. [34].

#### 2.4.4. Proteases Activity

Proteasome and cathepsin B and L activities in the liver and white muscle of fish were prepared and determined as described in Matias et al. [35]. Cathepsin activity was evaluated using 50 *μ*g of total protein per microplate well in both tissue samples and expressed by RFU per microgram of total protein. Proteasome activity was determined by using 25 and 75 *μ*g of total protein per microplate well in the liver and white muscle samples, respectively. Proteasome activity was expressed in milliunits per milligram of total protein in the sample.

#### 2.4.5. Microbiota Quantification on Fish Intestine

Fish terminal intestine (1 cm) was collected and homogenized with a pestle (Tissue Homogenizer, Ultraturrax) in a volume of 1 mL with sterile artificial seawater (*n* = 2 × 3) and sequentially diluted threefold and plated. Duplicates of 100 *μ*L of each dilution were spread on agar plates where tryptic soy agar (TSA from Merck, USA) was used to obtain the total number of aerobic bacteria, and thiosulfate-citrate-bile salts-sucrose agar (TCBS from OXOIDTM, USA) was used to isolate and count *Vibrionaceae*. Plates were incubated at 22°C for 7 days, and CFUs (colony-forming units) between 30 and 300 were counted after 2 and 7 days.

#### 2.4.6. Distal Intestine Histomorphology

When the trial ended, four fish were dissected on chilled trays, and the digestive tract was separated from the adjacent adipose and connective tissue. A section of the distal intestine (DI, distinguished from the anterior intestine by an enlarged diameter and darker mucosa) was sampled for histological analysis. Samples were rinsed in phosphate buffered saline (PBS), blotted dry with a paper towel, followed by immediate fixation in phosphate buffered formalin (4%, pH 7.4) for 24 h, and finally transferred to ethanol (70%) until further processing. Samples were processed and sectioned following standard histological protocols. Briefly, DI samples were processed in a tissue processor (Model Citadel 2000, Thermo Scientific, Nanjing, China), sectioned with a microtome (Model Jung RM 2035, Leica Instruments GmbH, Wetzlar, Germany) using standard histological techniques, and then stained with haematoxylin and eosin using an automatic slide (Model Shandon Varistain 24-4, Thermo Scientific, Nanjing, China). Double-blind evaluation of histological preparations was carried out, giving special attention to any inflammatory changes [36] such as changes in mucosal fold height (FH), width and cellularity of the lamina propria (LP) and submucosa (SM), number of intraepithelial lymphocytes (IELs), number of eosinophilic granular cells (EGCs), nucleus position, and supranuclear vacuolization within the enterocytes (ENT). A scale scoring system was used according to Couto et al. [37] with tissue scores ranging from 0 (normal) to 5 (highly modified). The overall value of histomorphological alterations was calculated by the average scores obtained for the parameters described above. Images were acquired with Zen software (Blue Edition; Zeiss, Jena, Germany).

#### 2.4.7. Muscle Cellularity

In this study, the muscle fibre area and density were examined in a 1 cm cross-section cut by the first ray of the dorsal fin, and a total of 6 fish per treatment were used. The methodology is detailed in Saavedra et al. [23].

### 2.5. Statistical Analysis

All data was tested for normality and homogeneity of variances (Shapiro-Wilk and Levene tests, respectively) and further analysed by one-way ANOVA considering the level of insect meal replacement in the diet. The multiple comparisons (Tukey) test was applied when significant differences were obtained and when equal variances were verified by Levene's test. For all data, statistical significance was tested at the 0.05 probability level. The comparison between diets considered tanks as the experimental unit. The data from intestinal histomorphology had to be analysed by the Kruskal-Wallis nonparametric test as it did not follow ANOVA assumptions. For the protease analysis, the Games-Howell test was applied when variances were unequal, and statistical tests were done using SPSS Statistics v21 software. For other digestive enzymes and intestine histomorphology, IBM SPSS software (v. 20; IBM, USA) was used to do the statistical analysis. Muscle cellularity statistical analysis was done using Statistica.

The specific growth rate (SGR) was calculated as SGR = ((ln *W*_*f*_–ln *W*_*i*_)/*t*)∗100, where *W*_*f*_ and *W*_*i*_ are the final and initial weights, respectively, and *t* is the trial duration in days.

Protein efficiency ratio (PER) was calculated as PER = (Final Biomass − Initial Biomass)/Crude Protein Fed.

Protein growth was determined as follows: Kg = (EXP(g) − 1) × 100 where *g* = (ln PROf–ln PROi)/*t*, and PRO = DW × %protein.

Protein retention (PRE) was calculated as the ratio between protein gain and protein intake multiplied by 100.

Feeding efficiency was calculated as the geight gain/feed consumption.

The Food conversion rate was calculated as food consumption/weight gain.

Fibre density was calculated as the number of fibres/muscle area. The muscle area was calculated using ImageJ.

## 3. Results

The survival rate was approximately 100% in all treatments. Differences were observed in the final weight (*p* = 0.02) and total length (*p* = 0.02) between meagre fed the control diet and the Ins20 diet (Table 2). Fish from the control treatment had higher weight, length, specific growth rate, and better food conversion ratio (FCR) compared to fish from the Ins20 treatment (Table 2). Meagre fed the Ins10 diet also showed differences from the Ins20 fish in terms of growth rate and FCR (Table 2). However, no significant differences were found between these treatments in the fish final weight or length. Significant differences were also registered for feed intake (*p* < 0.01) where a higher ingestion was found in fish fed the Ins10 diet, followed by fish fed the control diet, and, finally, fish fed the Ins20 diet.

The proximal composition of meagre was not affected by the diet (Table 3).

**Figure 1:**
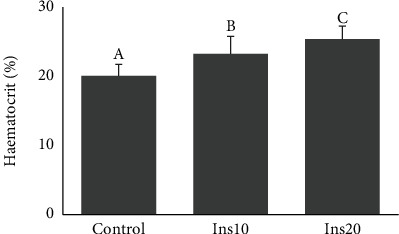
Meagre juvenile haematocrit fed three different diets with different incorporation of insect meal (IM). Control had no IM, Ins10 had 10% of IM incorporation in the diet and Ins20 had 20% of IM incorporation in the diet. Values are mean and standard deviation. Different letters represent significant differences for *p* < 0.05.

IM incorporation significantly affected the haematocrit of fish. Fish from the control group showed the lowest haematocrit (Figure 1). The heamatocrit increased with higher incorporation of IM (Figure 1).

**Figure 2:**
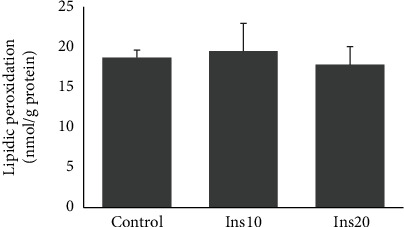
Lipid peroxidation (LPO) measured in red blood cells of meagre (*Argyrosomus regius*) fed three diets with different incorporation of insect meal (IM). Control had no IM, Ins10 had 10% of IM incorporation in the diet and Ins20 had 20% of IM incorporation in the diet. Values are mean and standard deviation.

No significant differences were observed between treatments for lipidic peroxidation of red blood cells (Figure 2).

Overall, digestive enzyme activities in meagre were not significantly affected by the incorporation of 10 or 20% IM in the diet when compared to the control treatment (Table 4). Trypsin-specific activity mean values ranged between 4.5 and 6.8 mU/mg protein among treatments. Acid phosphatase specific activity values ranged between 1.3 and 1.8, respectively, for control and Ins10, whereas total activity values ranged between 68.8 and 104.4 mU/mg protein, respectively, for Ins20 and Ins10. Aminopeptidase-specific activity was similar among treatments. However, significantly higher values of aminopeptidase total activity were observed for meagre fed the control and Ins10 diets when compared to meagre fed the Ins20 diet.

Regarding brush-border enzyme activities, meagre exhibited similar values both for alkaline phosphatase and aminopeptidase activities, regardless the type of diet. Meagre fed the control diet showed higher values for the alkaline phosphatase intestinal maturation index when compared with fish from Ins10 and Ins20. The same was not observed for the aminopeptidase intestinal maturation index.

**Figure 3:**
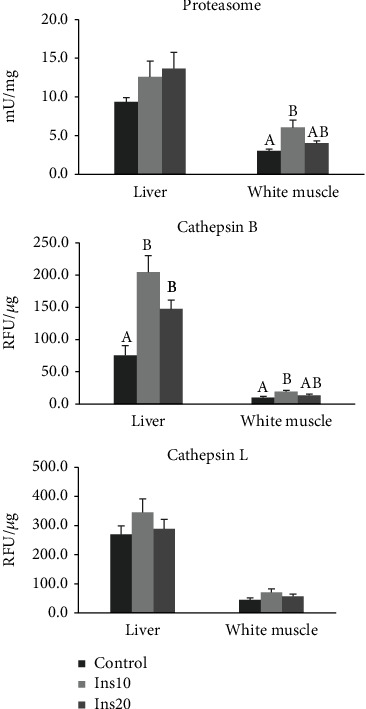
Forming Colonies Unities (FCU/ml) counted in TSA and TCBS medium from meagre (*Argyrosomus regius*) intestine fed three diets with different incorporation of insect meal (IM). Control had no IM, Ins10 had 10% of IM incorporation in the diet and Ins20 had 20% of IM incorporation in the diet. Values are mean and standard deviation. Different letters represent significant differences for *p* < 0.05.

Proteasome activity was significantly higher in the muscle of fish fed the Ins10 diet when compared to the control (*p* = 0.031) (Figure 3). However, no effect on the activity of this protease was observed in the liver of fish fed experimental diets (Figure 3). The activity of cathepsin B was significantly higher in the liver of fish fed the Ins10 and Ins20 diet than in the ones fed the control (*p* = 0.01 and *p* = 0.035, respectively) (Figure 3). In the muscle, the activity of this protease was only significantly higher for fish fed the Ins10 diet when compared with the control diet (*p* = 0.009). No differences were found in the activity of cathepsin L in liver or muscle (Figure 3).

**Figure 4:**
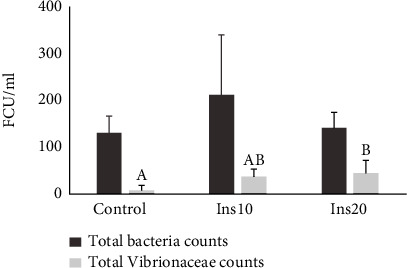
Proteasome activity (mU mg^−1^ total protein) and cathepsins B and L activity (RFU *μ*g^−1^ total protein) in the liver and white muscle of meagre juveniles diets with different incorporation of insect meal (IM). Control had no IM, Ins10 had 10% of IM incorporation in the diet and Ins20 had 20% of IM incorporation in the diet. Values are mean and standard error.

The introduction of IM did not affect the total bacterial count (Figure 4), but affected total *Vibrionaceae*, which showed significantly lower counts in the microbiota of meagre fed the control diet and higher in meagre fed the diet which contained 20% of IM (Figure 4).

**Figure 5:**
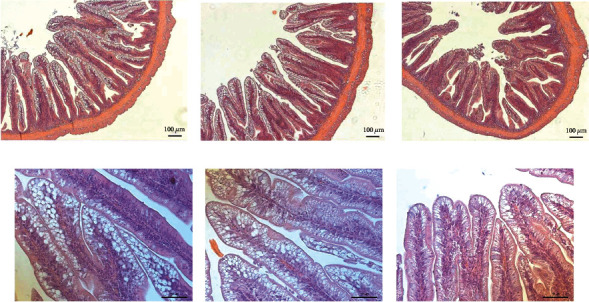
Histomorphological features of the distal intestine of meagre fed control diet without IM (a, d), a diet with 10% IM I(ns10) (b, e) and a diet with 20% IM (Ins20) (c, f).

The inclusion of IM in meagre diets had no marked effects on the distal intestine histomorphology (Table 5 and Figure 5). Fish from either treatment showed long, well-defined villous without fusion and normal lamina propria and submucosa thickness without increased cellularity. A medium-to-high frequency of intraepithelial leukocytes was observed in all dietary groups. Fish fed the control and INS10 diets presented enterocyte alterations with hypervacuolization and nucleus misplacement, while fish fed INS20 presented a more regular and normal-sized vacuolization along with a more aligned nucleus.

**Figure 6:**
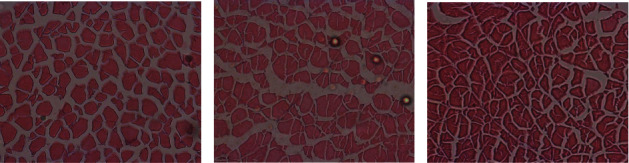
Muscle cellularity of meagre juveniles fed a control diet without IM (a), a diet with 10% insect meal (b) and a diet with 20% insect meal (c).

Muscle fibre area and density were not affected by the diet. The mean fibre area varied between 915 and 1550 *μ*m^2^ (Table 6 and Figure 6), and the muscle fibre density varied between 400 and 900 fibres per mm^2^.

## 4. Discussion

The potential of IM to replace FM has already been described for several fish species despite the presence of chitin which can affect diet digestibility [3, 19, 38]. In the current study, meagre final weight, total length, SGR, and FCR were significantly affected by the inclusion of 20% of IM. Similar results were obtained by Coutinho et al. [14] and Estévez et al. [39] for meagre of approximately the same weight (18 and 12 g, respectively) fed a diet with mealworms. Coutinho et al. [14] observed a decrease in body weight gain in meagre-fed diets with 10% or higher IM content. The same was reported for other species such as turbot and gilthead seabream, where the inclusion of IM, especially at higher levels, resulted in lower growth [40–42]. Nevertheless, this is not a general response for fish meal replacement, and there are studies showing successful inclusion of IM in species such as Atlantic salmon [43], European seabass [44], rainbow trout [17, 19], yellow catfish [45], tench [46], and zebrafish [47]. The constraints associated with IM digestion can, sometimes, be reflected in problems in the digestive tract of fish, including the intestine. Some studies have reported intestinal inflammation [18, 41, 44, 45]. In carp, the high IM inclusion was responsible for changes in intestine morphology that negatively affected fish growth [48, 49]. In the current study, the inclusion of IM in meagre diets caused minor changes in the distal intestine histomorphology. Meagre from all treatment showed long, well-defined villous without fusion. The only differences observed were in the enterocytes of fish from control and Ins10 which showed hypervacuolization and nucleus misplacement, whereas fish fed Ins20 had a more regular and normal-sized vacuolization along with a more aligned nucleus. This is consistent with Couto et al. [15] results who did not obtain significant changes in intestine histomorphology in meagerly fed IM, although some minor signs of inflammation were registered. In the current study, the presence of a higher percentage of *Vibrionaceae* in the treatments where IM was introduced suggests that inflammation could have occurred in the future. On the other hand, it is known that the IM fatty acid composition is usually rich in saturated fatty acids, namely, lauric acid, which protects gut health due to its intestinal anti-inflammatory, antibacterial [47, 50–53], and antiviral properties [50, 54–56]. In perch, Tran et al. [57] observed a reduction of *Lactobacillus* (*p* = 0.04) and *Streptococcus* in the fish gut when the fish were fed a diet with the incorporation of defatted *Tenebrio molitor*. Furthermore, chitin present in insects has the potential to improve fish immune system and promote their performance [9]. This suggests that although *Vibrionaceae* is present in a higher percentage, the IM could have provided a higher protection to the digestive tract from infection. This immunity protection against vibrio has been previously shown by Harikrishnan et al. [58] in kelp grouper, registering an increase in the haematocrit of fish fed diets supplemented with chitin. In the current study, it was also observed that there was an increase in the haematocrit with higher inclusions of IM in the diets (20% in the control and 25% in Ins20). Nevertheless, the haematocrit levels obtained in this study are lower than the ones obtained by Saavedra et al. [59] for smaller meagre (6.2 to 10.5 g with a haematocrit of 32% vs. 22%).

Lipidic peroxidation of cells can affect fish health, while antioxidant substances that can reduce oxidative stress play an important role. Stenberg et al. [60] obtained an increase in the levels of peroxidation products in Atlantic salmon fed diets containing IM. The authors thought that the higher concentration of certain heavy metals in the IM (such as cadmium) could induce a detoxification process, increasing lipidic peroxidation. In tench, diets incorporating *Tenebrio molitor* or *Hermetia illucens* promoted a high antioxidant capacity in the digestive tract and a decrease in lipid peroxidation compared to the control diet without IM [46]. However, in the present study, there were no differences in lipidic peroxidation in red blood cells between diets, suggesting that the level of IM replacement did not affect lipid peroxidation.

Meagre specific growth was affected by the inclusion of IM but was within the average SGR reported for meagre of this weight [23, 59] fed regular diets. The FCR of the Ins20 group was considerably higher than the ones usually registered for this species, which can vary between 0.8 and 1.5 [23, 59]. However, these differences did not affect the fish proximal composition. Coutinho et al. [14] observed a reduction in whole body protein, possibly resulting from the lower trypsin activity, and obtained lower lipase and total alkaline protease activity. In this study, only the total activity of aminopeptidase was affected by the inclusion of IM in the diet, which was lower in the Ins20 treatment. Aminopeptidase catalyses the cleavage of amino acids from proteins or polypeptides, so the amount of the amino acid pool available for protein metabolism can be affected. However, in this study, the differences in the activity of aminopeptidase showed no effect on the studied protein degradation pathways in the muscle of meagre, since the activity of proteasome and cathepsin B and L in the group of fish fed with the Ins20 diet showed no significant differences when compared to the other two treatments. Proteasome and cathepsin B activity in the muscle of meagre fed the Ins10 diet was twice that observed in the control group, suggesting that protein degradation was considerably higher in this group. The activity of cathepsin B in the liver was higher in both treatments with IM inclusion compared to the control. Chitin is an important component of insect meal that can reduce crude protein digestibility [13]. Basto et al. [61] obtained a percentage of chitin of 4.6% in yellow mealworms and 6.1% in black soldier flies. A higher percentage of chitin in the diet may reduce protein availability for cellular functions and thus less amount of protein to be degraded. This could explain the lowest growth obtained in the fish fed the Ins20 diet. The differences obtained in fish growth were not reflected in the meagre muscle fibre area or density.

In conclusion, yellow mealworm inclusion in a meagre diet is possible without affecting overall fish performance and possibly includes benefits in terms of distal intestine health. However, when the inclusion percentage is above 10%, fish growth and FCR can be reduced.

## Figures and Tables

**Table 1:** Ingredients and proximate composition of the experimental diets.

	Control	Ins10	Ins20
Ingredients (g/kg)			
Fishmeal	400	300	150
Fish protein concentrate	50	50	50
Insect meal (*Tenebrio* spp.)	0	100	200
Wheat gluten	150	150	150
Corn gluten	215	210	250
Wheat meal	70	58	45
Fish oil	102	102	106
Vitamin and mineral premix	10	10	10
Vitamin C (stay C35)	1	1	1
Antioxidant	2	2	2
Sodium propionate	1	1	1
Monocalcium phosphate	15	11	27
L-lysine	—	4	7
L-threonine	—	1	1
L-tryptophan	—	1	1
Composition (% DW)			
Protein	60.3 ± 0.3	60.4 ± 0.3	60.3 ± 0.1
Lipids	15.3 ± 0.0	17.2 ± 0.1	17.3 ± 0.1
Ash	10.9 ± 0.3	9.9 ± 0.0	8.2 ± 0.0
Energy (KJ)	23.0 ± 0.0	23.4 ± 0.0	23.8 ± 0.0

C: diet without IM, Ins10: diet with 10% incorporation of insect meal. Ins20: diet with 20% incorporation of insect meal.

**Table 2:** Meagre juvenile biometry at the beginning and end of the experimental trial which tested three different diets with different incorporations of insect meal (IM).

	Control	Ins10	Ins20
Survival (%)	100 ± 0.0	100.0 ± 0.0	98.8 ± 1.3
Wet weight (g)			
Initial	10.1 ± 0.2	10.1 ± 0.0	10.1 ± 0.1
Final	34.1 ± 1.3^a^	33.6 ± 0.8^ab^	27.8 ± 0.3^b^
Length (cm)			
Initial	10.2 ± 0.4	10.2 ± 0.4	10.2 ± 0.4
Final	15.6 ± 1.0^a^	15.3 ± 0.9^ab^	14.6 ± 1.0^b^
Biomass (g)			
Initial	811.0 ± 16.5	808.7 ± 1.7	807.3 ± 6.2
Final	2724.0 ± 104.0^a^	2685.0 ± 64.8^a^	2193.3 ± 8.5^b^
Biomass gain (g/day)	5.0 ± 0.1^a^	4.9 ± 0.2^a^	3.7 ± 0.0^b^
Growth (SGR)	2.6 ± 0.0^a^	2.6 ± 0.1^a^	2.2 ± 0.0^b^
Food intake (g)	2773.3 ± 22.9^a^	2836.3 ± 20.5^b^	2630.3 ± 18.9^c^
FCR	1.5 ± 0.1^a^	1.5 ± 0.1^a^	1.9 ± 0.0^b^

Control had no IM, Ins10 had 10% IM incorporation in the diet, and Ins20 had 20% IM incorporation in the diet. The values are the mean and standard deviation. SGR: specific growth rate; FCR: food conversion rate; BW: body weight. Different letters represent significant differences for *p* < 0.05.

**Table 3:** Meagre juvenile whole-body proximal composition at the beginning and end of the experimental trial which tested three different diets with different incorporations of insect meal (IM).

	Control	Ins10	Ins20
Protein (%)			
Initial	65.9 ± 0.9	65.9 ± 0.9	65.9 ± 0.9
Final	62.3 ± 2.3	63.6 ± 1.0	64.7 ± 1.7
Lipids (%)			
Initial	10.8 ± 0.1	10.8 ± 0.1	10.8 ± 0.1
Final	18.2 ± 0.7	17.8 ± 1.2	17.4 ± 0.4
Ash (%)			
Initial	16.8 ± 0.1	16.8 ± 0.1	16.8 ± 0.1
Final	14.7 ± 0.8	15.9 ± 1.7	15.3 ± 1.2
Dry matter (%)			
Initial	24.6 ± 0.1	24.6 ± 0.1	24.6 ± 0.1
Final	26.0 ± 0.2	25.9 ± 1.0	26.2 ± 1.2
Energy (kj/g)			
Initial	19.4 ± 0.2	19.4 ± 0.2	19.4 ± 0.2
Final	20.5 ± 0.2	20.5 ± 0.4	20.9 ± 0.3

Control had no IM, Ins10 had 10% IM incorporation in the diet, and Ins20 had 20% IM incorporation in the diet. The values are the mean and standard deviation.

**Table 4:** Pancreatic and intestinal digestive enzyme specific activities on the intestine of meagre juvenile fed three diets with different incorporations of insect meal (IM).

	Control	Ins10	Ins20
Specific activity			
Trypsin	4.7 ± 0.8	4.8 ± 1.3	6.8 ± 1.7
Amylase	0.9 ± 0.1	0.9 ± 0.1	1.1 ± 0.1
Acid phosphatase	1.3 ± 0.4	1.8 ± 0.2	1.5 ± 0.3
Alkaline phosphatase	115.0 ± 19.5	127.0 ± 14.0	128.6 ± 34.4
Amino peptidase	65.1 ± 2.0	68.6 ± 4.8	77.5 ± 15.1
Brush border			
Alkaline phosphatase	839.4 ± 78.4	793.0 ± 99.9	763.4 ± 227.4
Amino peptidase	452.5 ± 50.8	445.3 ± 9.9	446.6 ± 111.5
Total activity			
Trypsin	271.7 ± 58.2	257.3 ± 80.5	308.4 ± 26.6
Amylase	49.9 ± 7.7	49.3 ± 0.9	51.0 ± 7.2
Acid phosphatase	77.3 ± 20.4	104.4 ± 11.1	68.8 ± 22.3
Alkaline phosphatase	6689.4 ± 1000.7	7321.3 ± 1183.1	5836.5 ± 666.5
Amino peptidase	3846.5 ± 87.2^a^	3943.6 ± 224.0^a^	3539.6 ± 88.6^b^
Brush border			
Alkaline phosphatase	266.9 ± 30.7	227.9 ± 35.0	208.4 ± 28.4
Amino peptidase	140.9 ± 27.4	130.7 ± 37.2	119.9 ± 24.1
Maturation index			
Alkaline phosphatase index	436.9 ± 92.7^a^	228.7 ± 39.2^b^	296.3 ± 45.5^b^
Amino peptidase index	230.4 ± 93.7	133.3 ± 43.2	175.9 ± 19.9

Control had no IM, Ins10 had 10% IM incorporation in the diet, and Ins20 had 20% IM incorporation in the diet. The values are the mean and standard deviation. Specific activity is expressed by mU/mg protein, and total activity is expressed by U/segment. Maturation is the relation between the total activity of brush border enzymes and acid phosphatase. Different letters represent significant differences for *p* < 0.05.

**Table 5:** Histomorphological score of the distal intestine (DI) of meagre fed the experimental diets: mucosal fold height (FH), width and cellularity of the lamina propria (LP) and submucosa (SM), number of intraepithelial lymphocytes (IELs), number of eosinophilic granular cells (EGCs), nucleus position and supranuclear vacuolization within the enterocytes (ENT), and the average of all the parameter evaluated (mean score).

	Control	Ins10	Ins20
FH	1.0 ± 0.0	1.0 ± 0.0	1.1 ± 0.3
LP	1.1 ± 0.3	1.2 ± 0.4	1.2 ± 0.4
SM	1.7 ± 0.7	2.0 ± 0.6	1.8 ± 0.8
IEL	3.0 ± 0.7	3.4 ± 0.7	3.0 ± 0.7
EGCs	1.5 ± 0.4	1.8 ± 0.6	1.8 ± 0.4
ENT	2.3 ± 0.5^b^	2.4 ± 0.6^b^	1.7 ± 0.5^a^
Mean score	1.8 ± 0.2	2.0 ± 0.3	1.8 ± 0.2

Values are given as the mean ± standard deviation. Different letters represent significant differences for *p* < 0.05.

**Table 6:** Mean fibre and mean average of muscle fibres of meagre from control (no IM), Ins10 (10% IM), and Ins20 (20% IM) diets.

	Control	INS10	INS20
Mean fibre area (*μ*m^2^)	1162.9 ± 391.3	915.4 ± 252.6	1221.6 ± 164.4
Mean fibre density (n° fibres/mm^2^)	575.4 ± 168.4	722.7 ± 225.5	524.1 ± 103.9

## Data Availability

The data that support the findings of this study are available in Saavedra, Margarida (2022), “InsectMeal”, Mendeley Data, V1, doi: 10.17632/866rmhr3yz.1.
